# Characterizing Protracted Febrile Myalgia: Fasciitis and Vasculitis of the Fascia and Muscle as Novel Histopathological Features

**DOI:** 10.3390/jcm13247630

**Published:** 2024-12-14

**Authors:** José Hernández-Rodríguez, Lola Mestre-Trabal, Verónica Gómez-Caverzaschi, Olga Araújo, Magda Terenas, Ricardo Robaina, Julio Bolaños, Sergio Prieto-González, Jordi Antón, Jordi Yagüe, Xavier Tomás, Iban Aldecoa, Josep M. Grau

**Affiliations:** 1 Autoinflammatory Diseases Clinical Unit, Department of Autoimmune Diseases, Hospital Clínic de Barcelona, University of Barcelona, Institut d’Investigacions Biomèdiques August Pi i Sunyer (IDIBAPS), Center of the European Reference Network (ERN) for Rare Immunodeficiency, Autoinflammatory and Autoimmune Diseases (RITA), Spanish Center of the Centros, Servicios y Unidades de Referencia (CSUR) and Catalan Center of the Xarxa d’Unitats d’Expertesa Clínica (XUEC) for Autoinflammatory Diseases, 08036 Barcelona, Spain; 2 Department of Pediatric Rheumatology, Pediatric Immune Dysfunction Disease Study Group (GEMDIP), Institut de Recerca Sant Joan de Déu, Hospital Sant Joan de Déu, Center of the European Reference Network (ERN) for Rare Immunodeficiency, Autoinflammatory and Autoimmune Diseases (RITA), Spanish Center of the Centros, Servicios y Unidades de Referencia (CSUR) and Catalan Center of the Xarxa d’Unitats d’Expertesa Clínica (XUEC) for Autoinflammatory Diseases, 08950 Barcelona, Spain; 3 Department of Immunology, Hospital Clínic de Barcelona, University of Barcelona, Institut d’Investigacions Biomèdiques August Pi i Sunyer (IDIBAPS), Center of the European Reference Network (ERN) for Rare Immunodeficiency, Autoinflammatory and Autoimmune Diseases (RITA), Spanish Center of the Centros, Servicios y Unidades de Referencia (CSUR) and Catalan Center of the Xarxa d’Unitats d’Expertesa Clínica (XUEC) for Autoinflammatory Diseases, 08036 Barcelona, Spain; 4 Department of Radiology, Hospital Clínic de Barcelona, University of Barcelona, 08036 Barcelona, Spain; 5 Department of Anatomic Pathology, Hospital Clínic de Barcelona, Institut d’Investigacions Biomèdiques August Pi i Sunyer (IDIBAPS), University of Barcelona, 08036 Barcelona, Spain; 6 Department of Internal Medicine, Hospital Clínic de Barcelona, Institut d’Investigacions Biomèdiques August Pi i Sunyer (IDIBAPS), University of Barcelona, 08036 Barcelona, Spain

**Keywords:** autoinflammatory diseases, familial mediterranean fever, myalgia, protracted febrile myalgia, vasculitis, fasciitis, muscle biopsy

## Abstract

**Background**: Protracted febrile myalgia (PFM) is a rare but severe form of myalgia mainly occurring in pediatric patients with familial Mediterranean fever (FMF). PFM imaging and histopathological data remain scarce. **Objectives**: A comprehensive clinical, imaging, and histopathological characterization of PFM was performed by retrospectively analyzing a reference center cohort of adult patients with FMF and myalgia, and by a PubMed search of well-described cases with PFM. **Results**: Among 56 adults with FMF from our center, 32 (57.1%) experienced myalgia, which was generalized in 21 (37.5%) and affected lower limbs in 11 (19.6%) subjects. One (1.8%) patient suffered PFM, mainly affecting calves and Achilles tendons. From our patient’s detailed information and the data from 123 PFM cases reported in the literature, PFM was characterized as usually presenting with fever and severe generalized myalgia, with occasional involvement of lower legs and calves. It is mainly associated (in >90% of cases) with the pathogenic mutation M694V in the *MEFV* gene. Raised acute phase reactants and normal creatine kinase levels are constant. High glucocorticoid doses are useful in most patients, and sustained colchicine treatment protects from PFM recurrences. MRI may identify a variable degree of muscle inflammatory changes, especially subfascial and myofascial lesions with extension to tendinous structures. PFM histopathology is characterized by T-cell rich inflammatory infiltrates and vasculitis mainly involving the fasciae and myofascial areas, with a lower muscle extent. **Conclusions:** PFM can occur in children and adults and appears to be clinically manifested as fasciitis/tendinitis caused by a vasculitis of the fasciae rather than a major muscle vasculitis.

## 1. Introduction

Protracted febrile myalgia (PFM) is a severe form of myalgia typically occurring in patients with familial Mediterranean fever (FMF), a monogenic autoinflammatory disease caused by mutations in the *MEFV* gene, with a classical, although not constant, autosomal recessive inheritance [1,2]. In Eastern Mediterranean countries, the prevalence of FMF is as high as 1 case per 400 to 1000 inhabitants [3,4], while in Western Mediterranean populations such as Italy and Spain, FMF is considered a rare disease, with an estimated prevalence of 1 case per 45,000 to 120,000 inhabitants [5,6]. FMF is caused by a malfunction of the *MEFV* gene, which encodes pyrin, a protein involved in the inflammasome regulation. *MEFV* gene mutations lead to an inappropriate activation of the inflammasome and to the subsequent exaggerated production of proinflammatory cytokines, such as interleukin-1 (IL-1), which results in a redundant and unprovoked systemic inflammatory response [1]. FMF is typically characterized by recurrent, short-lasting episodes of fever, polyserositis, and joint inflammation. Additional features include myalgia, erysipela-like erythema, oral ulcers, and orchitis [1,2]. FMF symptoms usually occur in early childhood, and their presentation during adulthood is less frequent and also characterized by a milder presentation compared to children [7,8,9,10,11,12].

The main long-term FMF complications include amyloidosis, which has been described to occur in about 10–20% of untreated or uncontrolled patients, as well as chronic joint damage and accelerated cardiovascular disease [1,4,13,14]. Colchicine is the treatment of choice for FMF, since it controls disease activity, reduces the number of attacks, significantly minimizes the risk of amyloidosis, and improves long-term outcomes [14]. Colchicine provides anti-inflammatory effects by disrupting microtubule formation in leukocytes and by suppressing caspase activation within the inflammasome machinery, with the subsequent reduction in neutrophil chemotaxis and production of active IL-1, respectively [14]. Anti-IL-1 agents, including anakinra, canakinumab, and rilonacept, have also been demonstrated to be effective in patients with colchicine-resistant (and intolerant) FMF since their direct action blocks the overproduced IL-1 molecule (canakinumab and rilonacept) or its cellular receptor (anakinra) [14,15]. The utility of treating patients with FMF and colchicine and/or anti-IL-1 resistance (or intolerance) with tumor necrosis factor (TNF), IL-6, and Janus kinase (JAK) blocking agents is variable and limited to isolated case reports and small series [14].

Among FMF musculoskeletal manifestations, the incidence of any type of myalgia ranges from 2.5% to 58.8% [16,17,18,19,20,21,22,23]. Myalgia and exertional leg pain were both parameters included in the 1997 FMF diagnostic criteria delineated by Livneh et al. [24]. In 2000, Majeed et al. defined myalgia as pain or tenderness or both in the extremities in the absence of joint swelling and signs of underlying osteomyelitis and described three patterns of myalgia in a cohort of 264 children with FMF according to its severity, duration, and temperature rise [16]. These FMF-associated myalgia patterns included: (a) exercise-induced myalgia: it was characterized by episodes of severe myalgia, mainly at night, and low grade fever within 8 h of exercise, lasting for 1 to 3 days (the most common pattern, accounting for 81% of patients with myalgia); (b) Spontaneous myalgia: it consisted of mild-to-moderate myalgia without fever or precipitating factors, usually limited to a few hours (it occurs in 8% of patients with myalgia); and (c) PFM: it was defined as severe, disabling, generalized myalgia and high fever, lasting for 4–6 weeks if untreated (occurring in 11% of FMF cases with myalgia) [16]. In addition, PFM was usually accompanied by abdominal pain and joint inflammation, occasionally by transient purpura [16].

Although severe myalgia in FMF has been reported from 1974 [25,26,27], PFM was first recognized in 1994 by Langevitz et al. as a vasculitic condition in patients with FMF [28], and was subsequently reported by other authors as the most dramatic and severe clinical pattern of FMF-related myalgia [16,17,18,19,20,22,23,28,29,30,31,32,33,34,35,36,37,38,39,40,41,42,43,44,45,46,47,48,49,50,51,52,53,54,55,56,57]. This severe inflammatory condition, of still unknown etiopathogenesis, affects from 0.1% [17] to 11% [16] of patients with FMF, depending on the series [16,17,18,19,20,22,23,49,55]. In 2007, Kaplan et al. proposed a set of diagnostic criteria for PFM, based on a series of 15 pediatric patients with FMF and PFM, among whom 5 (33%) patients developed PFM as the first manifestation of FMF, and 93% of them had the mutation M694V in the *MEFV* gene (80% in homozygosis) [44] (Table 1).

In order to characterize PFM at demographic, clinical, genetic, biological, electrophysiological, imaging, histopathological, and therapeutic levels, the present study aimed to analyze the different patterns of myalgia, with special attention to PFM, in a series of adult patients with FMF from a reference center for autoinflammatory diseases. An additional comprehensive review of all well-described cases with PFM published in medical literature was also carried out.

## 2. Materials and Methods

### 2.1. Patients, Variables Collected, and Ethical Considerations

Data from all patients consecutively diagnosed with FMF and followed at the Adult Clinical Unit of Autoinflammatory Diseases, Department of Autoimmune Diseases, Hospital Clínic de Barcelona, from January 2010 to December 2023, were retrospectively reviewed. All patients met the 2019 Eurofever/Paediatric Rheumatology International Trials Organisation (PRINTO) classification criteria for FMF [58]. Patients who did not meet these criteria at disease presentation and those in whom an alternative diagnosis was achieved during the follow-up were excluded.

Sex, ethnicity, family history of periodic fever, and age at disease onset and at diagnosis were the demographic variables collected in FMF patients. Clinical manifestations recorded included fever, abdominal pain, chest pain, skin lesions, oral aphthae, ocular involvement, odynophagia, cervical adenitis or lymphadenopathies in other territories, hepatomegaly, splenomegaly, and articular and muscle involvement. Myalgia was defined as pain or tenderness or both, and its pattern described as localized (bilateral or unilateral in upper or lower limbs) or generalized (all muscular groups in all extremities). The location was assessed as mostly proximal or mostly distal or was referred (when present) to the muscle group mainly or exclusively affected (e.g., lower limbs or calves). Laboratory markers recorded included C-reactive protein (CRP), erythrocyte sedimentation rate (ESR), hemoglobin levels, and leukocyte and platelet counts during attack and attack-free periods, as well as creatinine, glomerular filtration rate, urinalysis, and creatine kinase (CK) levels. Variants found in the *MEFV* gene were also recorded.

The response to the drugs used was also collected and considered as: (a) Complete, when total control of symptoms and inflammatory parameters was achieved with the used medication, without requiring additional drugs; (b) Partial, when the treatment was associated with any clinical improvement (not complete) that allowed continuing taking the drug, with or without the addition of new medications; and (c) No response, defined as the absence of effect leading to the discontinuation of the drug.

This retrospective descriptive study was approved by the Research Ethics Committee of the Hospital Clínic de Barcelona (HCB/2018/1165). Patients’ information was dissociated prior to analysis, and all procedures were performed in accordance with the ethical principles expressed in the 2013 Declaration of Helsinki.

### 2.2. Literature Review

An exhaustive PubMed search of FMF and PFM in English-written articles was carried out for case reports and case series published until 2023 by using the terms “familial Mediterranean fever”, “myalgia” and “protracted febrile myalgia”. Those missed but relevant references included in the selected articles were manually searched. Only articles clearly describing FMF and PFM features were selected, and all the information identified was also analyzed.

### 2.3. Statistical Analysis

All results were assessed according to categorical variables, absolute and relative frequencies, and expressed in numbers, and percentages. Continuous variables were expressed as means and standard deviations (SD) for descriptive purposes.

## 3. Results

### 3.1. FMF and PFM in Our Adult Reference Center

Fifty-six adult patients with FMF, 31 (55.4%) females, were followed in our center during a mean of 12 years. Half of them had a family history of periodic fever, which was confirmed as FMF affecting a relative by genetic study in 17 (30.4%) patients. Disease onset occurred during adulthood in 29 (52%) patients. A mean (SD) delay until disease diagnosis was of 8.9 (9.5) years. The most frequent manifestations were fever (mean [SD] temperature 39.3 [1] °C) in 51 (91.1%) patients, followed by abdominal pain in 47 (83.9%), arthralgia/arthritis in 41 (73.2%), myalgia in 32 (57.1%), and pleuritic chest pain in 22 (39.3%) patients. Among patients presenting myalgia, pain was generalized or affected proximal muscles in 21 (37.5%) patients and predominated in lower limbs in 11 (19.6%) cases. In the latter group, PFM was diagnosed in one (1.8%) patient with FMF. The mean demographic, clinical, and laboratory features at disease presentation are illustrated in Table 2.

Fifty-four (96.4%) patients were treated with colchicine, with complete, partial, and no response in 20 (37%), 25 (46.3%), and 9 (16.7%), respectively. Colchicine was discontinued in 3 (5.6%) of patients due to gastrointestinal intolerance, mostly diarrhea. Glucocorticoids were used on demand during FMF flares in 19 (33.9%) patients, with better control of symptoms in all of them. Fourteen (25%) patients received biological agents, mainly IL-1 blockers (either anakinra or canakinumab), with partial or complete response in 10 (71.4%) of them. Four (7.1%) patients refractory to previous drugs received tocilizumab with good response in 3 (75%) of them.

### 3.2. Our Patient with Protracted Febrile Myalgia

#### 3.2.1. Clinical Presentation

A Spanish Caucasian 27-year-old man who had initiated with periodic attacks of fever and abdominal pain since the age of 8 years was diagnosed with FMF at the age of 20 years. The analysis of the *MEFV* gene showed the heterozygous compound M694I/R202Q, with no pathogenic variants in other genes tested (*TNFRSF1A*, *MVK*, *NLRP3*, *NOD2*, *ADA2*, and *PSTPIP1*). Colchicine was started at variable doses (1–1.5 mg/day) with good control of the attacks. Following a decrease in colchicine dose to 0.5 mg/day, he presented a prolonged flare of 6 weeks duration with weight loss (of 4 kg) and profuse sweating, also accompanied with progressive and severe myalgia in the proximal muscles of upper limbs and lower limbs, more evident in the calves, with inability to stand and walk. Physical examination was remarkable for diffuse abdominal pain upon deep palpation and incapability to walk due to intense calves’ pain. Bilateral tenderness and sensitive pain on palpation of the gastrocnemius muscle, Achilles tendons, and extensor toe tendons of the feet were noted. Other muscle groups were not painful.

#### 3.2.2. Complementary Tests

- Laboratory and Electromyogram Results

Laboratory tests showed increased phase acute reactant levels (CRP 7.6 mg/dL and ESR 97 mm/h), normal leukocytes (8.9 × 10^9^/L) and platelet counts (214 × 10^9^/L), slightly reduced hemoglobin levels (124 g/L), and normal kidney function, urinalysis, hepatic enzymes, CK (28 UI/L), and lactate dehydrogenase levels. Extensive microbiologic cultures and serologies were negative. An electromyogram (EMG) did not find myopathic or neuropathic abnormalities.

- Muscle magnetic resonance imaging

**Figure 1:**
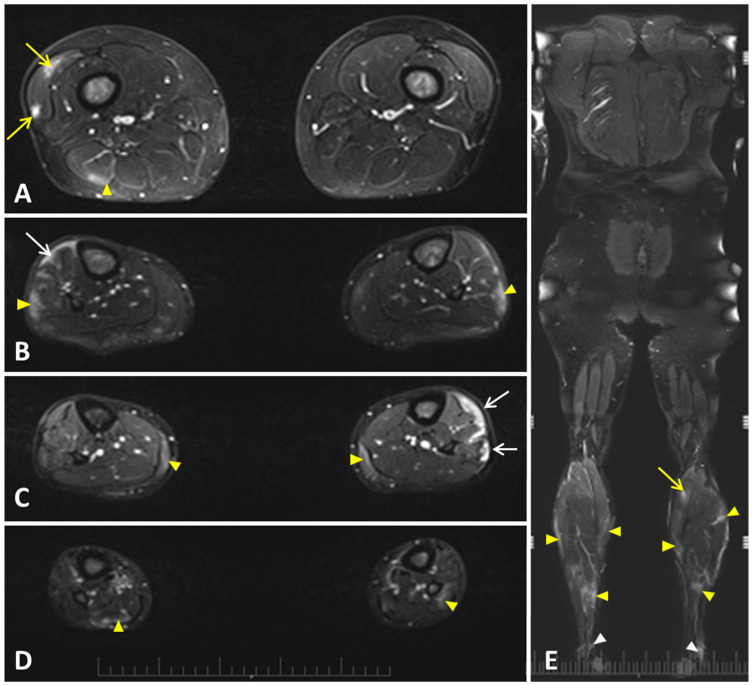
Cranio-caudal axial STIR-weighted whole body magnetic resonance imaging (WBMRI) of the thighs (**A**) and legs (**B**–**D**), and a coronal section of the entire body (**E**) of our patient with familial Mediterranean fever and protracted febrile myalgia. Three different patterns of hyperintense areas suggesting edema were detected: (1) Tubular-rounded foci within the muscle of the thigh (long yellow arrows) involving the right vastus lateralis (**A**) and the left gastrocnemius (**E**), with hiperintensity of the medullary bone (**A**); (2) Subfascial pseudocollections (long white arrows) involving right deep fascia-tibialis anterior and extensor digitorum longus muscles (**B**) and the same level of the opposite leg, including the leg fibularis muscle (**C**); and (3) Subtle, cotton-like miofascial foci (yellow arrowheads) affecting the right biceps femoral muscle (**A**), bilateral fibularis muscles (**B**,**E**), bilateral medial head of gastrocnemius (**C**,**E**), and right soleus muscle and left fibularis mucle (**D**,**E**). Hyperintense signal of both Achilles tendons (white arrowheads) is also observed (**E**).

A whole body magnetic resonance imaging (WBMRI) showed hyperintense lesions as tubular-rounded foci within the right vastus lateralis muscle in the thigh and the gastrocnemius in the leg, as well as more evident subfascial pseudocollections of the deep fascia-tibialis anterior and extensor digitorum longus and leg fibularis muscle, and cotton-like myofascial foci affecting several leg muscles, including the head of gastrocnemius and soleus muscle, with inflammatory involvement of the Achilles tendons (Table 3 and Table 4 and Figure 1).

- Histopathology of the fascia and gastrocnemius muscle

**Figure 2:**
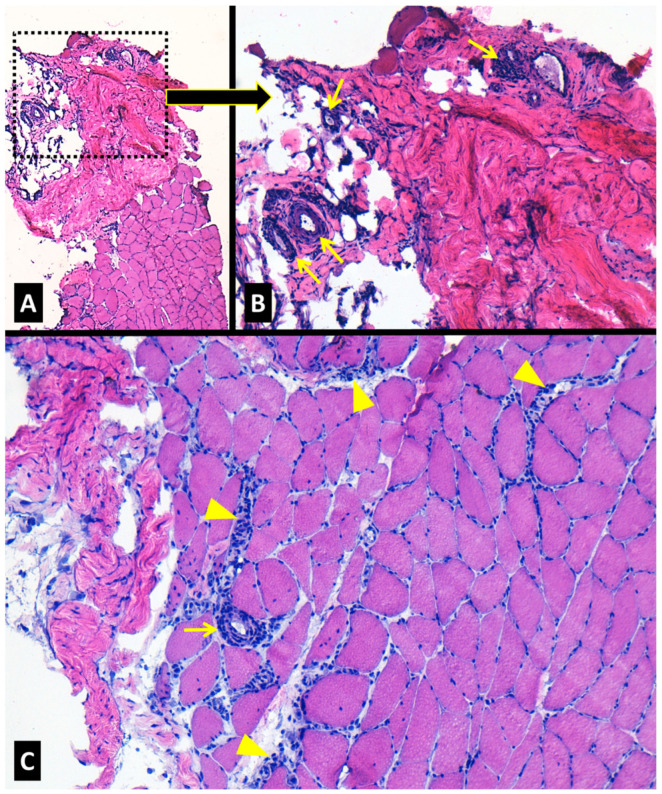
Histopathologic features of the gastrocnemius muscle biopsy of our patient with familial Mediterranean fever and protracted febrile myalgia. (**A**) Muscle without evident inflammation and adjacent peripheral myofascial tissue with inflammatory infiltrates. (**B**) Detailed magnification from the myofascial area (square in (**A**)) showing a small-vessel vasculitis (arrows). (**C**) Muscle with scarce inflammatory cells in endomysium (arrowheads) and a small vessel vasculitis (arrow) in areas close to the peripheral myofascial tissue (arrow). *Hematoxylin-eosin staining*; *Original magnification* ×*40* (**A**), ×*100* (**B**,**C**).

**Figure 3:**
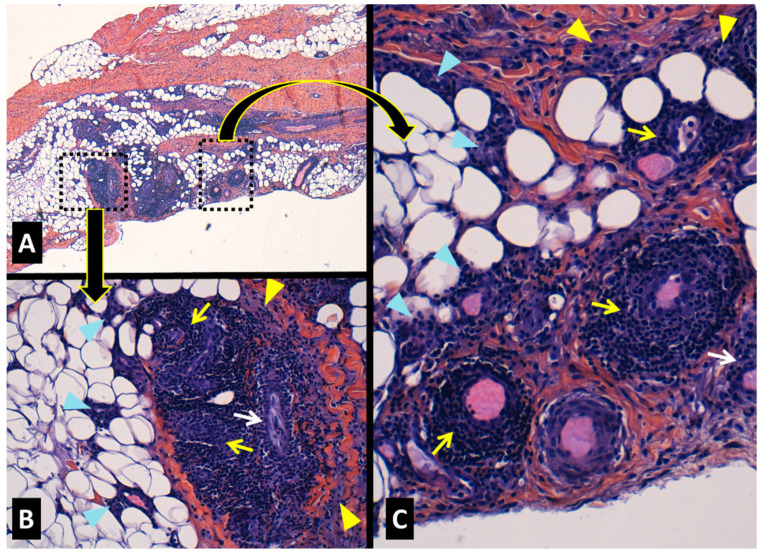
Histopathology of the fascia (contiguous to the gastrocnemius muscle) of our patient with familial Mediterranean fever and protracted febrile myalgia, showing a lymphocyte predominant fasciitis with vasculitis and reactive fibrosis (**A**). Selected areas ((**B**,**C**) from squares in (**A**)) showing a dense lymphocyte predominant inflammation in small-sized arteries (yellow arrows), small veins (white arrows), connective tissue (yellow arrowheads), and small vessels within the adipose tissue (blue arrowheads). *Hematoxylin-eosin staining*; *Original magnification* ×*40* (**A**), ×*200* (**B**,**C**).

A biopsy of the medial head of the right calf muscle and its contiguous fascia was performed prior to glucocorticoid therapy. The muscle revealed scarce lymphomonocytic endomysial infiltrates and small-vessel vasculitis in isolated small arteries close to peripheral myofascial tissue, without remarkable inflammation in deeper areas. Peripheral fibrous tissue compatible with myofascial areas also showed a small vessel vasculitis (Figure 2A–C). The fascia displayed a non-necrotizing vasculitis of small-sized arteries and small venules within the connective and adipose tissue with variable reactive fibrosis of the fascia with interspersed inflammatory lymphomonocytic infiltrates (Figure 3A–C).

**Figure 4:**
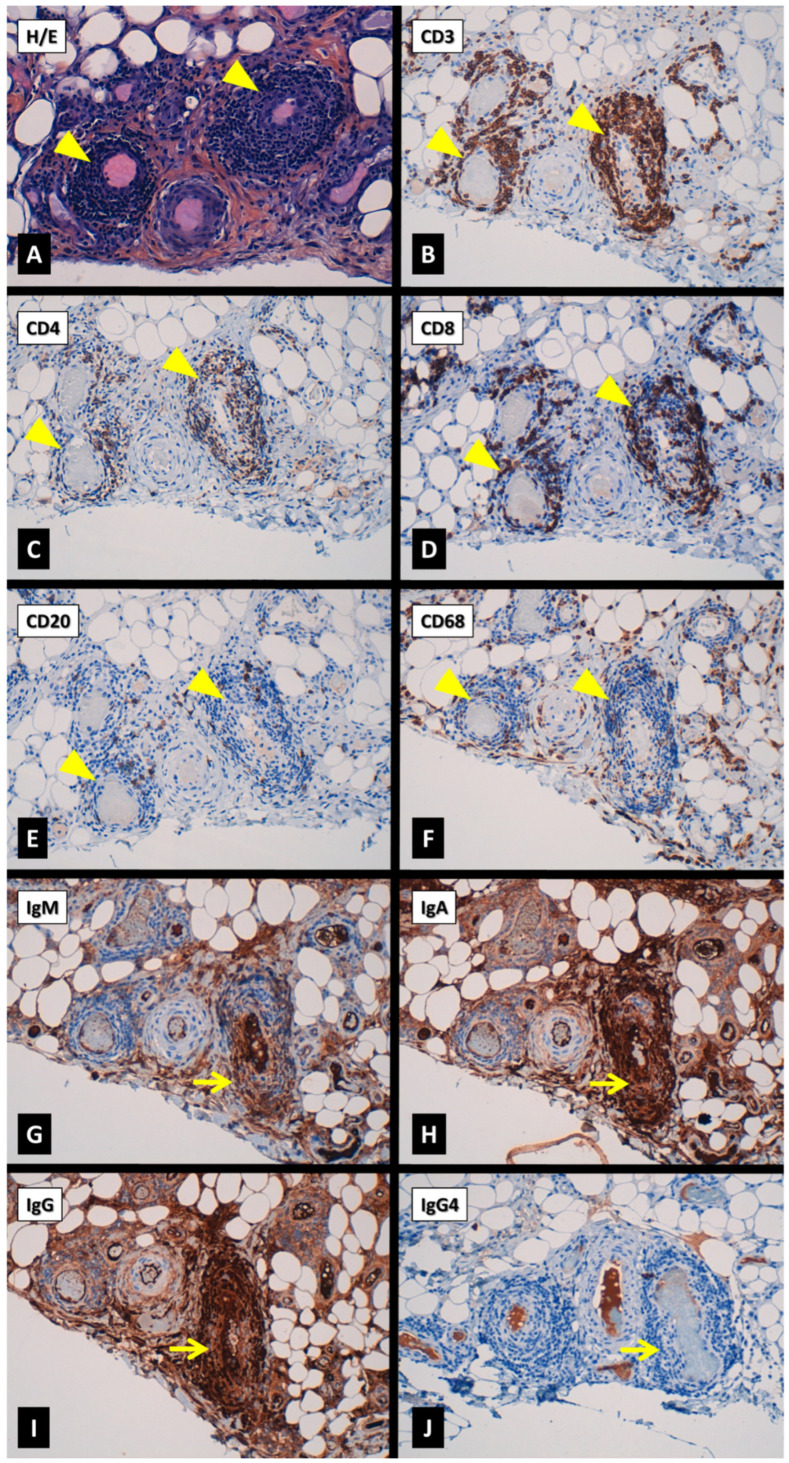
Immunohistochemical profiling of the vascular inflammatory infiltrates of the fascia; sequential sections (**A**–**F**) of two inflamed small arteries (arrowheads). Vasculitic infiltrates showed a clear predominance of T lymphocytes (mainly CD3+) and a slight prevalence of CD8+ over CD4+ T lymphocytes. Macrophages (CD68+) were more frequent in the connective tissue, being occasional in the vessels, while B lymphocytes (CD20+) were scarce. Immunohistochemistry assessing immunoglobulins (Ig) within the inflamed vessel walls (**G**–**J**) (yellow arrows) showed a mild expression of IgM (**G**) and strong staining for IgA (**H**) and IgG (**I**), with no remarkable expression of IgG4 (**J**). *Hematoxylin-eosin staining* (**H**/**E**) in (**A**); *Original magnification* ×*200* (from (**A**–**J**)).

The immunohistochemical profiling of the perivascular infiltrates in fascia highlighted a T CD3+ predominant infiltrate with a slight predominance of T cytotoxic (CD8+) over T helper (CD4+) lymphocytes, with occasional macrophages (CD68+) (more frequent in the connective tissue) and isolated B lymphocytes (CD20+) (Figure 4A–F). The assessment of immunoglobulins (Ig) showed a marked predominance of IgA and IgG, with occasional IgM cells and almost no IgG4 cells (Figure 4G–J). Congo red staining was negative in muscle and fascia biopsies.

#### 3.2.3. Treatment Used and Outcome During Follow-Up

During admission, colchicine dosage was increased from 1 to 1.5 mg/day, and prednisone was administered at 0.5 mg/kg/day for 2 weeks with subsequent tapering until its discontinuation in 4 months. All symptoms progressively improved, and after 10 days, the patient was discharged with slight calf pain on palpation and normalized laboratory values.

After 10 years of follow-up, the patient has presented several FMF attacks with fever and abdominal pain, usually after periods of intense physical exercise and irregular intake of colchicine. He also experienced two episodes of slight myalgia in lower limbs, mainly calves, but symptoms were abrogated by adding prednisone at 0.5 mg/d for 5–7 days with subsequent tapering until cessation in 3 to 6 weeks. The patient is currently on colchicine 1.5 mg/day and tolerates well all types of exercise.

### 3.3. PFM in Medical Literature

Descriptions of PFM in the PubMed search were found in 123 patients [16,18,27,28,30,31,32,33,34,35,36,37,38,39,40,41,42,43,45,46,47,48,49,50,51,52,53,54,56,57]. Demographic characteristics, clinical manifestations at disease presentation, laboratory markers, genetic results, EMG, imaging and histological features, and response to the treatments administered are illustrated in Table 3. Sixty-six (53.7%) patients were females, and 121 (98.4%) belonged to south-eastern Mediterranean countries. Mean (SD) age at FMF and PFM symptoms onset was 10.5 (8) and 15 (9.4) years, respectively. PFM was part of the FMF onset in 35.2% of patients, and 16 (13%) of them had an adult PFM presentation.

Among 76 (61.8%) individuals with available data about myalgia features, it was bilateral in 71 (93.4%) patients (and 6.6% unilateral), 46 out of 61 (75.4%) patients had generalized myalgia, and 18 (29.5%) and 6 (9.8%) of them complained of predominantly proximal and distal muscle pain, respectively. In 24 of 34 (70.6%) and 8 of 13 (61.5%) patients with information in this regard, the pain was remarkable in the lower limbs and calves, respectively.

FMF manifestations concomitantly present with PFM included fever in 110 (89.4%) patients, abdominal pain in 88 (71.5%), arthralgia/arthritis in 45 (36.6%), and vasculitic rash in 42 (34.1%) cases [16,18,27,28,30,31,32,33,34,35,36,37,38,39,40,41,42,43,45,46,47,48,49,50,51,52,53,54,56,57]. Mean (SD) CRP and ESR values were 17.6 (16.2) mg/dL and 91 (23) mm/h, respectively. Mean (SD) leukocyte and platelet counts were 16 (4) × 10^9^/L and 505 (125) × 10^9^/L, respectively [16,18,27,28,30,31,32,33,34,35,36,37,38,39,40,41,42,43,45,46,47,48,49,50,51,52,53,54,56,57]. CK levels were normal in all patients but one, in whom raised CK levels were reported to be due to a pharmacological effect [31]. Among the 92 patients with genetic study, M694V was the *MEFV* gene mutation more frequently found in patients with PFM (*n* = 85 [92.4%]; as homozygous compound in 61 [66.3%] [18,28,30,31,32,34,35,36,38,40,42,44,46,47,49,50,51,52,54,57], as heterozygous compound in 18 [19.6%] patients [32,37,38,45,48,49,50,52,54], and as a single heterozygous mutation in 6 [6.5%] [32,44,47]). Other variants different from M694V, either in homozygosity or in heterozygous compounds, were found in 7 (7.6%) cases [18,40,41,43,46,53,57]. EMG was performed in 38 patients [16,27,28,31,33,34,35,37,44,48,52,54,55] and a myopathic pattern was detected in 20 (52.6%) of them [16,28,31,44,54,55].

Muscle MRI results were reported in 11 (8.9%) patients with PFM [32,35,41,43,45,47,48]. Muscular edema suggesting myositis was identified in 8 (72.6%) subjects [32,43,45,47], inflammatory changes of the fascia were described in 2 (18.2%) patients [41,43], and 2 (18.2%) MRIs were reported as normal [35,48]. In one MRI, myositis and fasciitis features were both present [43], and in another MRI reporting myositis, additional inflammatory features were described in the subcutaneous fat tissue and the distal part of the gastrocnemius muscle prior to the musculocutaneous junction of the Achilles tendon [45]. Among the 17 (13.8%) muscle biopsies performed, 14 (82.4%) samples were normal [16,32,33,52,54,55], and 3 (17.6%) biopsies showed inflammatory infiltrates affecting either (mildly) muscle [27] or (more evidently) fascia [43,45]. Cutaneous biopsies were reported in 18 (14.6%) patients, with perivascular mononuclear infiltrates and leukocytoclastic vasculitis in 7 (38.9%) [54,55,59] and 4 (22.2%) [28,34,39,51] samples, respectively. Seven (38.9%) skin biopsies were normal [34,38,45,51,54,55]. Table 4 included the description of muscle/fascia MRI and biopsy findings in patients with PFM identified in the medical literature.

Information about treatment was available in 116 (94.3%) patients with PFM. Glucocorticoids, usually oral prednisone at doses of 1–2 mg/kg/day (or equivalent) or intravenous methylprednisolone (at higher doses) during several days (independently if patients were treated with colchicine or not), were clearly useful in improving PFM symptoms in 91 of 94 (96.8%) patients who received them [16,18,28,31,32,33,34,35,37,38,39,40,41,42,44,46,48,49,50,51,52,53,54,55,56,57]. A rapid tapering with prednisone cessation in 2 to 12 weeks was reported by several authors [16,33,37,39,40,41,48,49,50,57], with no repeated myalgia episodes if patients were later on maintained colchicine. The use alone of colchicine or NSAIDs was effective in 5 (4.3%) [16,27,45,55] and 14 (12.1%) [32,44,49] patients previously not taking these drugs, respectively. Anakinra controlled PFM symptoms in 5 (4.3%) patients, either in those with previous partial or no effect to colchicine or glucocorticoids [36,57], or when administered alone as the first option [47]. Subcutaneous tocilizumab at 162 mg per week was also effective in a patient with PFM with no response to glucocorticoids [43]. A spontaneous resolution of severe myalgia after 6–7 weeks without any treatment was reported in 2 (16.2%) patients [44] (Table 3).

## 4. Discussion

Although FMF is known to be a pediatric disease, FMF manifestations may also start during adulthood, as occurred in our cohort of adult patients. In this sense, FMF and other monogenic autoinflammatory diseases share several differences between pediatric and adult presentations. While children usually present with more intense or severe manifestations, develop more frequently long-term complications such as secondary amyloidosis, and pediatric-onset diseases are often caused by pathogenic variants, adult patients tend to have milder symptoms, lower risk of developing amyloidosis, and a genetic profile with a higher frequency of variants of uncertain significance, non-confirmatory genotypes, and postzygotic variants [7,8,9,10,11,12].

Myalgia is a relatively common manifestation in FMF, ranging from 2.5% to 58.8% [16,17,18,19,20,21,22,23], and PFM (the most severe form of myalgia) is developed by 0.1% to 11% of patients with FMF [16,17,18,19,20,22,23,49,55]. Although most of the series and single cases analyzing the presence of myalgia and PFM are based on pediatric populations, in our adult FMF series, any type of myalgia and PFM similarly occurred in 57.1% and 1.8% of patients, respectively. Myalgia tended to be generalized in the majority of our adult patients, with predominant involvement of lower limbs in around a third of subjects with myalgia. In patients with PFM, muscle pain has been more frequently described as generalized, with predominant proximal (more than distal) involvement. Lower limbs and calves were also remarkably involved in cases in which detailed data about muscle groups affected was reported. Our patient also presented with proximal pain in the four extremities but also with severe and disabling pain in the calves and Achilles tendons.

In PFM, acute phase reactants, such as CRP and ESR, are constantly high, and CK is typically in the normal range. At the genetic level, PFM is more frequently associated (in >90% of cases) with the presence of the M694V variant, either in homozygosity or as a heterozygous compound with other pathogenic mutations [30,32,34,35,36,37,38,40,42,44,47,48,49,50,51,52,54,57]. However, PFM has been less frequently reported in individuals with M694V as a single heterozygous variant [32,38,44,47,54], occasionally in patients with variants different from M694V (in homozygosity or in heterozygous compounds) [18,40,41,43,46,53,57], and in a patient carrying the R202Q polymorphism in a heterozygous compound with M694V [48]. Similarly, our patient carried the heterozygous compound M694I/R202Q in the *MEFV* gene. In this regard, the role of R202Q is still unclear since while it has been considered a polymorphism not involved in the pathogenesis of FMF [58,60], other studies are postulating the contribution of R202Q in FMF inflammatory manifestations [61,62,63,64].

EMG may be normal or depict myopathic abnormalities in about half of patients. Data about muscle MRI in patients with PFM are scarce to date. Although some MRI did not report muscular abnormalities [35,48], other authors have reported muscle involvement with edema suggesting myositis [32,43,45,47], inflammatory changes of the fascia [41,43], or both [43], with additional inflammation of the distal part of the gastrocnemius muscle prior to the musculocutaneous junction of the Achilles tendon and the subcutaneous fat tissue [45]. Interestingly, the muscular WBMRI of our patient with PFM showed inflammatory changes within several muscles in the thigh and the leg and more prominent subfascial lesions and cotton-like myofascial foci in several muscles of the legs, such as the gastrocnemius and soleus muscles, with involvement of the Achilles tendons.

Muscle histopathology in PFM has been described as normal in the majority of the few biopsies reported [16,32,33,52,54,55], and only three samples have detected inflammatory infiltrates in the muscle [27] or the fascia [43,45]. A combined muscle and fascia biopsy in our patient demonstrated the presence of inflammatory mixed lymphomonocytic (mainly T cells) infiltrates in both tissues. Indeed, the inflammation was more intense at the myofascial (fibrocollagenous) muscular area and fascia, in which small- and medium-sized vessel vasculitis was concomitantly present, particularly in the fascia. Cutaneous inflammation as perivascular mononuclear infiltrates and leukocytoclastic vasculitis adjacent to the inflamed muscular areas has also been reported to occur in some patients with PFM [28,34,39,51,54,55,59].

Treatment with glucocorticoids, such as prednisone (or equivalent) at doses no lower than 0.5 mg/kg, or even higher doses of prednisone or methylprednisolone (in cases with partial effect to previous prednisone doses), has been highly effective in ameliorating and resolving the severe PFM manifestations [16,18,28,31,32,33,34,35,37,38,39,40,41,42,44,46,48,49,50,51,52,53,54,55,56,57]. Most patients are able to taper and stop glucocorticoids in a short period of time, usually within 3 months [16,33,37,39,40,41,48,49,50,57]. To avoid PFM recurrences, it is important to initiate colchicine (in cases not treated until the PFM episode) or to increase or adjust colchicine at therapeutic doses (usually at 0.3 mg/day) in those patients already receiving it. A lactose-free and anti-diarrhea diet will contribute to achieving therapeutic colchicine doses [14].

Several inflammatory diseases have to be considered within the differential diagnosis of PFM. Inflammatory myopathy with abundant macrophages (IMAM) is a rare condition involving muscles and skin. Although its clinical presentation differs from that of PFM, IMAM is characterized by muscle inflammatory infiltrates with a predominance of macrophages (CD68+) and few CD3+ T cells, mainly CD4+ T helper cells [65,66,67,68]. Dense macrophagic infiltrates involving the fascia have also been described in some patients with IMAM [65,69,70]. However, no vasculitis has been concomitantly reported in any of them [65,66,67,68,69,70]. IMAM has been differentiated from dermatomyositis [65,66,67,68], macrophagic myofasciitis [65] and has been concomitantly described with Still’s disease [69]. Although IMAM has not been reported to occur in patients with FMF or tumor necrosis factor receptor-associated periodic syndrome (TRAPS), several variants in *MEFV* and *TNFRSF1A* genes have been detected in patients with IMAM [70]. Anyway, in order to clearly differentiate PFM from IMAM in patients with FMF, an immunohistochemical study of the cell composition within the infiltrates is warranted.

Because PFM is a condition with vasculitis affecting the muscles and fasciae, differential diagnosis with polyarteritis nodosa (PAN), IgA vasculitis (in patients with concomitant cutaneous purpuric lesions), and Behçet’s disease has to be additionally performed in these patients, indeed when these three primary systemic vasculitides are more often presented in patients with FMF than in the general population [71,72]. However, the fact that the glucocorticoids may be discontinued in around 3 months in most patients with no recurrences of PFM makes PFM clearly a different condition from PAN or IgA vasculitis, since these primary systemic vasculitides tend to require glucocorticoids for a longer time and often additional immunosuppressive agents, usually in severe or refractory cases [73,74].

PFM etiopathogenesis remains unclear. However, even though lymphocyte clonality has not been analyzed in our case, the absence of B cells and the predominance of T cells within the vasculitic infiltrates in the fascia might indicate a T cell response towards a recognized (but unknown) antigen. The good response to glucocorticoids and the ability to be able to discontinue them without new PFM flares in the majority of patients reported, together with the previous descriptions of spontaneous remissions of PFM, support the hypothesis of a reactive and transient inflammatory response causing PFM.

In agreement with Langevitz et al., who initially described PFM as a vasculitic condition in patients with FMF [28], our case also sheds light on the histopathology of this severe FMF manifestation, a protracted condition, and recognizes PFM as a T cell-rich vasculitis producing severe pain in the muscles, fasciae, and tendons (as the final structures of the muscular fasciae), which is caused by a profuse involvement of the fasciae, and in lesser degree the muscle, as described in our patient and in previous PFM reports [27,43,45]. The fact that vasculitis affects the vessels in the fascia probably explains the high frequency of muscle biopsies with normal results previously reported in the literature [16,32,33,52,54,55]. Therefore, in order to better achieve an appropriate diagnosis when PFM is suspected, a muscle biopsy together with a fascia sample must be obtained. Of note, the fascia is thicker and easy to obtain as a differentiated tissue in lower limbs (as in our patient).

The current study has several limitations due to its retrospective design and the small sample of patients with FMF, with only one of them presenting with PFM. However, the strengths of our study are based on the analysis of myalgia in a population of adult patients with FMF that shows a similar proportion of PFM to that described in FMF pediatric series and the detailed imaging and histopathologic characterization of the inflammatory lesions involving the fascia and muscle for the first time in medical literature. Of note, to the best of our knowledge, our reported case was the first patient with FMF and PFM described in Spain.

## 5. Conclusions

PFM is a rare manifestation of FMF in pediatric and adult patients presenting as a severe and limiting generalized myalgia, with predominant involvement of lower legs, calves, and Achilles tendons in some cases. It is associated with pathogenic variants in the *MEFV* gene, mainly M694V. Raised acute phase reactants (CRP and ESR) and normal values of CK are constant. The prognosis of PFM is generally good, with complete recovery and a low probability of recurrences if treated promptly and appropriately. Glucocorticoids, such as prednisone, at initial doses of 0.5 mg/kg/day or higher for a total period of at least 3 months, are useful in most patients. Maintenance with therapeutic doses of colchicine protects from recurrences of PFM. MRI may identify a variable degree of inflammatory changes in the affected muscles, but subfascial and myofascial lesions with extension to tendinous structures are the most relevant findings. Histopathology of PFM is characterized by diffuse T-cell rich inflammatory infiltrates and vasculitis mainly involving the fasciae and myofascial areas and the muscles to a lower extent. Therefore, PFM appears to be clinically presented as fasciitis/tendinitis caused by a vasculitis of the fasciae rather than a major muscle vasculitis. However, these histopathological findings, which could indeed be useful to propose a new set of diagnostic or classification criteria, should be prospectively explored and confirmed in larger studies.

## Figures and Tables

**Table 1:** Proposed diagnostic criteria for protracted febrile myalgia (PFM) by Kaplan et al. (2007) [44].

	Obligatory Criteria	Supporting Criteria
**(1)**	***Familial Mediterranean fever (FMF)***. Prior clinical and/or genetic evidence of FMF or familial history of FMF	Homozygous or heterozygous compounds with the M694V mutation
**(2)**	***Myalgia***. Bilateral, of the lower and/or upper limbs, characterized by severe muscle pain and tenderness with physical disability, in the presence of normal muscle enzyme levels	Elevated acute phase reactants: ESR ≥ 80 mm/h, CRP ≥ 1.5 mg/dL
**(3)**	***Duration of symptoms***. Persistence of the myalgia for ≥ 5 days.	Fever ≥ 38 °C
	***Exclusion criteria***	
	Polyarteritis nodosa	

**Table 2:** Main demographic, clinical, and laboratory features in our series of 56 patients with familial Mediterranean fever (FMF).

Characteristics	FMF Patients N (%)
**Number of patients**	56
**Demography**; *n (%)*	
Women/men	31 (55.4)/25 (44.6)
Mediterranean ethnicity *	52 (94.5)
Family history of periodic fever	28 (50)
**Disease presentation**	
Adult onset (≥16 years old); *n (%)*	29 (52)
Age at symptoms onset (years); *mean (SD)*	19.5 (13.7)
Age at diagnosis (years); *mean (SD)*	28.4 (14.3)
Diagnosis delay (years); *mean (SD)*	8.9 (9.5)
Follow-up time (years); *mean (SD)*	12 (10.6)
**Clinical features**; *n (%)*	
Fever	51 (91.1)
Abdominal pain	47 (83.9)
*Peritonitis*	20 (42.6)
*Previous appendectomy*	12 (25.5)
Chest pain	27 (48.2)
*Pleuritic*	22 (81.5)
Pleural effusion	7 (12.5)
Pericarditis	16 (28.6)
Pericardial effusion	6 (10.7)
Arthralgia	37 (66.1)
Arthritis	19 (33.9)
Myalgia	32 (57.1)
*Generalized (proximal and distal)*	21 (37.5)
*Lower limbs predominantly*	11 (19.6)
*Protracted febrile myalgia*	1 (1.8)
Cutaneous rash **	12 (21.4)
Ocular manifestations **	9 (16.1)
Odynophagia	15 (26.8)
Oral aphthae	11 (19.6)
Cervical adenopathy	8 (14.3)
Lymphadenopathy **	11 (19.6)
Hepatomegaly	2 (3.6)
Splenomegaly	5 (8.9)
**Laboratory values**; *mean (SD)* ^#^	
CRP (mg/dL)	
*Attacks*	14.7 (7.5)
*Intercritical periods*	2.3 (3)
ESR (mm/1st hour)	
*Attacks*	40 (36)
*Intercritical periods*	7.6 (6.8)
Leukocyte (attacks) (×10^9^/L)	10 (5)
Platelets (attacks) (×10^9^/L)	251 (107)
Hemoglobin (attacks) (g/L)	126 (19.8)

Abbreviations: CRP = C-reactive protein; ESR= Erythrocyte sedimentation rate; SD = Standard deviation. * Mediterranean ethnicity includes occidental populations [Spanish (*n* = 40) and Moroccan (*n* = 3)] and eastern Mediterranean populations [Georgian (*n* = 1), Armenian (*n* = 5), Turkish (*n* = 1), Israelian (*n* = 1) and Arabian (*n* = 1)]. One patient was from England and two from South America, but one of them with a known Spanish ancestry. No data available from one patient. ** Cutaneous rashes included erysipeloid rash, erythematous, desquamative, and bullous lesions, and erythema nodosum; Ocular manifestations included conjunctivitis, episcleritis, uveitis, vitritis, and optic neuritis; Lymphadenopathy included the involvement of submandibular, axillary, mediastinal, abdominal, mesenteric, retroperitoneal, and inguinal lymph nodes. ^#^ From 33 patients with available data during attacks and from 54 patients during intercritical periods.

**Table 3:** Main characteristics of protracted febrile myalgia (PFM) described in the 123 patients with familial Mediterranean fever (FMF) collected from the medical literature and those data from our patient.

Characteristics	Patients with PFM N (%)	PFM *Present Case*
**Number of patients**	123	1
**Demography;** *n (%)*		
Women/men	66 (53.7)/57 (46.3)	Man
Mediterranean area *	121 (98.4)	1
**Disease presentation**		
Mean age at FMF symptoms onset (years); *mean (SD)*	10.5 (8)	8
Mean age at PFM diagnosis (years); *mean (SD)*	15 (9.4)	27
Adult PFM onset; *n (%)*	16 (13)	1
PFM as part of FMF onset; *n (%)*	32/91 (35.2)	0
**Clinical features**; *n (%)*		
Fever	110 (89.4)	1
Abdominal pain	90 (73.2)	1
Arthralgia/arthritis	41 (33.3)	1
Purpuric rash	27 (22)	0
Erysipeloid/other rashes	14 (11.4)	0
Pleural/pericardial pain/effusion	10 (8.1)	0
Scrotal swelling/pain	3 (2.4)	0
Myalgia ^#^		
*Bilateral*	71/76 (93.4)	1
*Unilateral*	5/76 (6.6)	0
*Generalized (proximal and/or distal)*	46/61 (75.4)	1
Proximal (mostly)	18/61 (29.5)	0
Distal (mostly)	6/61 (9.8)	0
*Lower limbs predominantly*	24/34 (70.6)	1
*Calf muscles predominantly*	8/13 (61.5)	1
**Laboratory (during attacks)**		
CK (normal values); *n (%)* ^§^	122 (99.2)	1
CRP (mg/dL); *mean (SD)*	17.6 (16.2)	7.6
ESR (mm/1st hour); *mean (SD)*	91 (23)	97
Leukocyte (×10^9^/L); *mean (SD)*	16 (4)	9
Platelets (×10^9^/L); *mean (SD)*	505 (125)	214
***MEFV* gene study**; *n (%)*		
M694V in homozygous compounds	61/92 (66.3)	0
M694V in heterozygous compounds	18/92 (19.6)	0
M694V as a single heterozygous variant	6/92 (6.5)	0
Other (non-M694V) variants ^⁋^	7/92 (7.6)	1
**Electromyogram**; *n (%)*		
Myopathic pattern	20/38 (52.6)	0
Normal pattern	18/38 (47.4)	1
**Magnetic resonance imaging**; *n (%)*		
Myositis pattern	8/11 (72.6)	1
Fasciitis changes	2/11 (18.2)	1
Normal	2/11 (18.2)	0
**Histopathology**; *n (%)*		
Muscle/fascia biopsy		
*Inflammatory infiltrates* ^†^	3/17 (17.6)	1
*Normal/no inflammation*	14/17 (82.4)	0
Skin biopsy		
*Perivascular mononuclear cell infiltrates*	7/18 (38.9)	0
*Leukocytoclastic vasculitis*	4/18 (22.2)	0
*Normal*/*no inflammation*	7/18 (38.9)	0
**Response to treatment**; *n (%)*		
Glucocorticoids ^‡^	94/116 (81)	1
Colchicine (alone)	5/116 (4.3)	0
NSAIDs (alone)	14/116 (12.1)	0
Anakinra	5/116 (4.3)	0
Tocilizumab	1/116 (0.9)	0

Abbreviations: CK = Creatine kinase; CRP = C-reactive protein; ESR = Erythrocyte sedimentation rate; NSAIDs = Non-steroidal anti-inflammatory drugs. * Countries belonging to the Mediterranean area where patients with PFM were reported cases include Turkey, Jordan, Israel, Iraq, Arabia, and Morocco. Two patients were from Japan, and our patient was from Spain. ^#^ From patients with available/detailed information about myalgia location. ^§^ One patient had elevated CK due to myopathy induced by cyclosporine and colchicine administration during 2 months after kidney transplant [31]. ^⁋^ Genetic compounds not including the M694V mutation were reported in 7 patients with PFM: homozygous M680I (*n* = 2) [18,57], homozygous E148Q [53], homozygous E148Q and P369S-R408Q [41], M694I heterozygous [43], A744S heterozygous [46], and E148Q/R761H [40] compound (*n* = 1 each). ^†^ Inflammatory infiltrates are described either in muscle, musculotendinous junction, or fascia (see Table 4 for details). ^‡^ Glucocorticoids were effective in 91 of 94 (96.8%) patients in whom they were used alone or in combination with colchicine (colchicine was added when patients were not receiving the drug previously).

**Table 4:** Description of muscle/fascia MRI and biopsy findings in patients with familial Mediterranean fever (FMF) and protracted febrile myalgia (PFM) identified in the medical literature and in our center *.

Author/Year (Reference)	Patients with PFM	Age at PFM Onset (years)	Sex (F/M)	Muscular MRI	Muscular Biopsy
Schapira D et al. 1988 [27]	1/1	22	1/0	Not performed	Collagen deposit in the muscular interstitium and few inflammatory cells (fibroblasts, macrophages, and few mast cells)
Kotevoglu N et al. 2004 [45]	1/1	13	1/0	Non-specific edema of the subcutaneous fat tissue and the distal part of the medial gastrocnemius muscle prior to the musculocutaneous junction of the Achilles tendon (highly suggestive of an inflammatory lesion)	Scarce non-specific inflammatory infiltration with leukocytes, lymphocytes and eosinophils in the cutaneous and subcutaneous tissue and muscular fascia
Fujikawa K et al. 2014 [41]	1/1	22	0/1	Thickening of the fascia on STIR sequences, suggesting inflammation of the fascia (rather than in the muscle fibers) and fasciitis as main cause of PFM	Not performed
Mercan R et al. 2014 [47]	2/2	41/44	2/0	Remarkable muscle edema in calf muscles, consistent with PFM (performed in 1 patient)	Not performed
Honda N et al. 2021 [43]	1/1	20	0/1	Nonspecific muscular edema and fascia thickening	Biopsy of the cutaneous/subcutaneous tissue, muscle and fascia with infiltration of macrophages and lymphocytes involving the fascia (suggesting that PFM is a type of fasciitis rather than vasculitis)
Aviran N et al. 2022 [32]	5/5	10 (6–16)	3/2	Muscular edema with high intensity signal on STIR (compatible with myositis), without signs of fasciitis or vasculitis (performed in the 5 patients)	Normal (performed in 1 patient).
Our present case	1/56	27	1/0	STIR-WBMRI with hyperintense lesions as tubular-rounded foci within the right vastus lateralis muscle, subfascial pseudocollections of the deep fascia-tibialis anterior and extensor digitorum longus and leg fibularis muscle, and cotton-like miofascial foci affecting several leg muscles including the head of gastrocnemius and soleus muscle, with involvement of the aponeurotic proximal origin of the Achilles tendon	Inflammatory infiltrates and lymphocyte predominant small- and medium-sized vessel vasculitis, denser in the fascia and myofascial areas, and occasional small-vessel vasculitis in the muscle. Immunohistochemistry with predominance of T lymphocytes (mainly CD3+), followed by CD8+ and CD4+ T lymphocytes in the inflamed vessel walls. Occasional macrophages (CD68+) and scarce B cells (CD20+) were also present.

Abbreviations: STIR = Short-time inversion recovery; WBMRI = Weighted whole body magnetic resonance imaging. * Six additional patients with FMF and PFM were reported to have normal muscle biopsies [one case each in references [16,33,52,55] and four biopsies in reference [54]; and two additional muscle MRIs were reported as normal [35,48].

## Data Availability

The data supporting the findings of this study are available from the corresponding author upon reasonable request.
